# Combined impact of *CHCHD10* p.Gly66Val and three other variants suggests oligogenic contributions to ALS

**DOI:** 10.3389/fneur.2025.1438207

**Published:** 2025-03-18

**Authors:** YiYing Wang, YuXin Mi, Hui Wang, JingSi Jiang, Le Mao, YanXi Heng, XiaoGang Li, Min Deng

**Affiliations:** ^1^Institute of Medical Innovation and Research, Peking University Third Hospital, Beijing, China; ^2^Daxing Research Institute, University of Science and Technology Beijing, Beijing, China; ^3^Department of Neurology, Peking University Third Hospital, Beijing, China

**Keywords:** amyotrophic lateral sclerosis, *CHCHD10*, oligogenic pathogenesis, whole-genome sequencing, genotype-phenotype analysis

## Abstract

**Introduction:**

Amyotrophic lateral sclerosis (ALS) is a severe neurodegenerative disease characterized by a progressive loss of motor neurons and muscle atrophy. Genetic factors are known to play important roles in ALS and concomitant presence of rare variants in ALS patients have been increasingly reported.

**Methods:**

In order to explore the genetic variants in ALS patients within the context of oligogenic inheritance and to elucidate the clinical heterogeneity observed in these patients, we conducted whole-genome sequencing on 34 familial ALS (FALS) probands.

**Results:**

In one proband, we identified a *CHCHD10* p.Gly66Val variant, along with three additional variants: *UNC13A* p.Leu1034Val, *SUSD1* p.Trp704Ser, and *SQSTM1* p.His359del. This patient exhibited a slow disease progression and a prolonged survival duration, consistent with the clinical features of ALS patients with *CHCHD10* variants. This suggests that the *CHCHD10* p.Gly66Val variant may play a predominant role in shaping the patient's phenotype, while the other variants may primarily contribute to ALS occurrence.

**Discussion:**

Variants in *CHCHD10* have been found in ALS and other neurodegenerative diseases, exhibiting significant clinical variability. However, the combinatorial effect of *CHCHD10* and other ALS-related gene variants has not been fully studied. Our findings suggest that the combined impact of these four variants contributes to this patient's ALS phenotype, distinguishing it from other, less severe neuromuscular disorders associated with *CHCHD10* mutations. Overall, this study further supports the oligogenic pathogenic basis of ALS and offers new insights into understanding the intricate clinical presentations associated with *CHCHD10* variants.

## 1 Introduction

Amyotrophic lateral sclerosis (ALS) is a severe neurodegenerative disease that affects both upper and lower motor neurons, leading to gradual degeneration and loss of these cells. Patients with ALS exhibit highly heterogeneous clinical manifestations marked by progressive muscular atrophy, and often die from respiratory failure within 2–4 years after the symptom initiation (1). Typically, ALS can be classified as the familial (FALS) or sporadic (SALS) form. Research has revealed that heredity plays a significant role in ALS, especially in familial cases. However, patients from different ethnicities exhibit a distinct genetic basis. In the Chinese ALS population, the most prevalent mutation causing ALS is located in the *SOD1* gene, representing around 21.9% of familial cases and 1.90% of sporadic cases, followed by *FUS* and *TARDBP*, accounting for 6.3 and 3.1% of FALS patients, respectively (2). Currently, despite the growing identification of pathogenic genes linked to ALS, its etiology remains largely unknown.

The *CHCHD10* gene encodes a protein containing coiled-coil-helix-coiled-coil-helix domains, which is implicated in maintaining mitochondrial cristae morphology and mitochondrial morphological remodeling (3). Since 2014, many mutations in *CHCHD10* have been discovered in ALS patients, solidifying its status as a definitive ALS gene (4). Subsequent studies have also unveiled a wide range of *CHCHD10*-related disorders, including late-onset spinal motor neuronopathy (LOSMoN/SMAJ) (5), axonal Charcot-Marie-Tooth disease type 2 (CMT2) (6), Alzheimer's disease (7), and myopathy (8). Consequently, patients with the same *CHCHD10* variant can exhibit a wide variety of clinical phenotypes, suggesting the complexity of the underlying pathogenesis mechanism, possibly involving other contributing factors.

Recent studies have also unveiled the oligogenic pathogenic pattern of ALS, where multiple mutations in ALS-related genes can be identified within the same patient, contributing collectively to disease onset (9–11). This model arose due to the observation of incomplete penetrance in FALS cases with a clear autosomal dominant inheritance pattern, suggesting the potential involvement of additional genetic factors (12). Several studies have reported on the prevalence of oligogenic carriers across different ALS cohorts. For example, Naruse et al. identified multiple variants in ALS-related genes through whole-exome sequencing (WES) in 7/56 FALS probands and 8/87 SALS patients (9). Additionally, Scarlino et al. discovered that 17% of ALS patients in an Italian cohort carried multi-gene variants through next-generation sequencing (NGS) (13). The combined effect of these variants contributes to the onset of the disease and often influences phenotypic characteristics, such as a more severe clinical phenotype, which may include earlier age of onset (9, 14) and poorer prognosis (10, 13). Therefore, the oligogenic model provides new insights into the clinical heterogeneity observed in ALS patients. While the number of newly identified genes related to ALS is increasing, mutation screenings remain predominantly centered around common ALS-associated genes, with limited researches employing comprehensive mutation screening across all potential ALS-related genes, especially in Chinese population. In light of this, there is an increasing need for thorough genetic screening of ALS patients.

Therefore, we conducted whole-genome sequencing (WGS) on 34 Chinese FALS probands to gain a thorough understanding of the genetic landscape of these patients. Given the intricate clinical phenotypes observed in patients carrying *CHCHD10* variants, we specifically focused on this gene in this study. We explored whether *CHCHD10* variants co-occurred with additional variants in the other 112 ALS-associated genes, aiming to provide further insights into the potential role of oligogenic pathogenesis in ALS.

## 2 Materials and methods

### 2.1 Participants

In total, 127 probands diagnosed with familial amyotrophic lateral sclerosis (FALS), meeting the criteria for definite or probable ALS as per the EI criteria (15), were recruited from the Department of Neurology at Peking University Third Hospital during 2003 and 2018. Additionally, some of the family members were also enrolled for subsequent segregation analysis. Written informed consent for involvement in both clinical and genetic research was obtained from all participants during their hospital visits. This project received approval from the Peking University Third Hospital Institutional Ethics Committee (No. IRB00006761-L2010055), and was conducted in accordance with the principles of the Declaration of Helsinki.

Detailed clinical features information of these patients was collected, including age of onset, initial symptoms, family history, and medication usage. Additionally, regular telephonic follow-ups were conducted. Disease severity was evaluated using the ALS Function Rating Scale (ALSFRS).

We first screened these 127 FALS probands for common mutations in the four most prevalent ALS genes (*SOD1, FUS, TARDBP*, and *C9orf72*). Mutations in *SOD1, FUS*, and *TARDBP* were identified using polymerase chain reaction (PCR) and Sanger sequencing. The repeat length of the pathogenic *C9orf72* G4C2 repeat expansion was examined by repeat-primed PCR combined with fragment length analysis using fluorescently labeled primers as previously reported (16). Patients with negative results in these four genes were considered more likely to harbor rare or novel variants in other ALS-related genes. To further elucidate the mutation status of these patients, we conducted whole-genome sequencing on probands from 34 pedigrees. The selection of these probands for WGS was based on a comprehensive evaluation of factors including the completeness of clinical phenotypes and follow-up information, the quality of preserved DNA samples, and the availability of DNA from additional family members.

### 2.2 DNA preparation

DNA samples were extracted from 5 ml peripheral blood collected from the participants using a QIAamp^®^ DNA Blood Mini Kit (Qiagen, Valencia, CA, USA) and quantified using the Qubit 3.0 fluorometer (Life Technologies, Paisley, UK). Genomic DNA was broken up into DNA fragments between 50 and 800 bp on Covaris E220 (Covaris, Brighton, UK), which were further narrowed down using AMPure XP beads (AGENCOURT) to between 100 and about 300 bp for construction of the sequencing library by NanoBalls (DNB) technology.

### 2.3 Sequencing and data analysis

**Figure 1:**
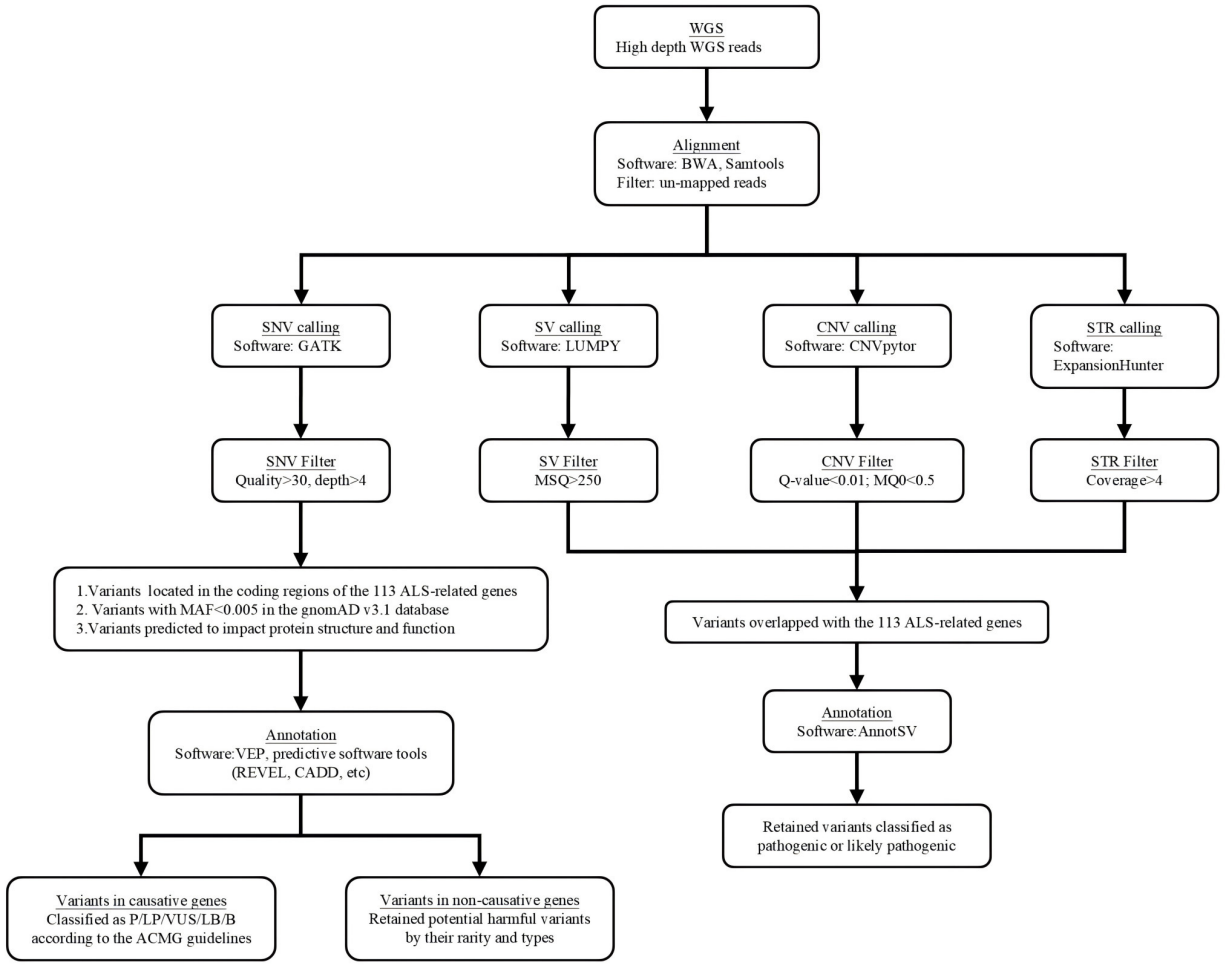
The filtering and screening process of whole genome sequencing analysis.

Whole-genome sequencing was performed using the BGISEQ-500 protocol provided by BGI (Beijing Genomics Institution), incorporating DNA nanoball and probe-anchor synthesis technologies for library construction, quality control, pooling, and sequencing. The filtering and screening process for whole-genome sequencing analysis is illustrated in Figure 1. SOAPnuke (version 2.0.1) was utilized for filtering raw reads, removing those containing a fraction of gaps (*N*) or low-quality bases (*Q* < 20) exceeding a threshold of >0.1. The resulting clean reads were aligned to the human reference genome (hg38) using the Burrows-Wheeler Aligner (version 0.7.15), and the alignment summaries were generated by samtools (version 1.15.1).

Different filtering criteria were applied based on variant types. SNVs and INDELs were identified using the Genome Analysis Tool Kit (v3.6) and filtered with the following QC criteria: read depth < 4, mapping quality < 55, variant quality < 30, and genotype quality < 30. SVs were detected with LUMPY (v0.2.13) and SVTyper (v0.1.4), and excluded if: split-read and paired-end counts < 10%, mean sample quality < 150, deletion size < sequencing library insert size, or copy number estimates (CNVnator) >0.5 for deletions or < 1.5 for duplications. CNVs were detected using CNVnator (v0.4, 100 bp bins), merged with bedtools (v2.30.0, 1 bp overlap), and excluded if *Q-*value > 0.05, zero map quality reads > 50%, gaps > 0, or size < 1,000 bp, or > 100,000 bp. Repeat expansions were called with ExpansionHunter (v5.0.0), excluding variants with quality < 30 or coverage depth < 10×.

### 2.4 Variant identification

To comprehensively analyze the potential disease-related variants in these patients, we systematically collected ALS-related genes from OMIM (https://www.omim.org/), PubMed (https://pubmed.ncbi.nlm.nih.gov/), Google Scholar (https://scholar.google.com/), the MGI database (https://www.informatics.jax.org/), and ALSoD (https://alsod.ac.uk/). Aiming to obtain a balance between the breadth of gene inclusion and the feasibility of data analysis, we included all 113 genes that have been previously associated with ALS in cellular or familial studies (Supplementary Table S1). Variants with MAF > 0.005 in the gnomAD v4.1.0 database were excluded. Variants located in the coding region of these 113 ALS-associated genes and were predicted to impact protein structure and function were retained for further evaluation. For causative genes, we scored and categorized the variants based on the American College of Medical Genetics (ACMG) guidelines. For variants located on non-causative genes, we filtered rare variants through frequency (MAF < 0.005 in gnomAD database), selected potentially harmful variants by variant type, and ranked the variants in conjunction with the results from software tools that predicts harmfulness. We used VEP (version 107) software, which integrates tools like SIFT (17) and PolyPhen-2 (18) for predicting the impact of variants on protein function. Additionally, we employed other *in silico* prediction tools—CADD (19), FATHMM (20), MutationTaster (21), M-CAP Score (22), and REVEL (23)—to further evaluate their pathogenic potential. PhastCons (24) and phyloP (25) scores were also used to assess the evolutionary conservation of these variants. Variants predicted to be harmful by multiple tools were given priority under equivalent conditions.

Annotation and Ranking of Human Structural Variations (AnnotSV) was employed to provide comprehensive annotations and scores for SVs, CNVs, and STRs, with scores ranging from 1 to 5 corresponding to P, LP, VUS, LB, and B. Variants meeting two conditions were preserved: those classified as pathogenic or likely pathogenic by AnnotSV, and those where the variant's genomic region overlapped with any of the 113 ALS-associated genes.

All variants identified were validated through PCR and Sanger sequencing. Based on the reference genomic sequences of the human genome from GenBank at NCBI, primer pairs were designed for the candidate loci (Supplementary Table S2).

## 3 Results

### 3.1 Genetic findings

**Figure 2:**
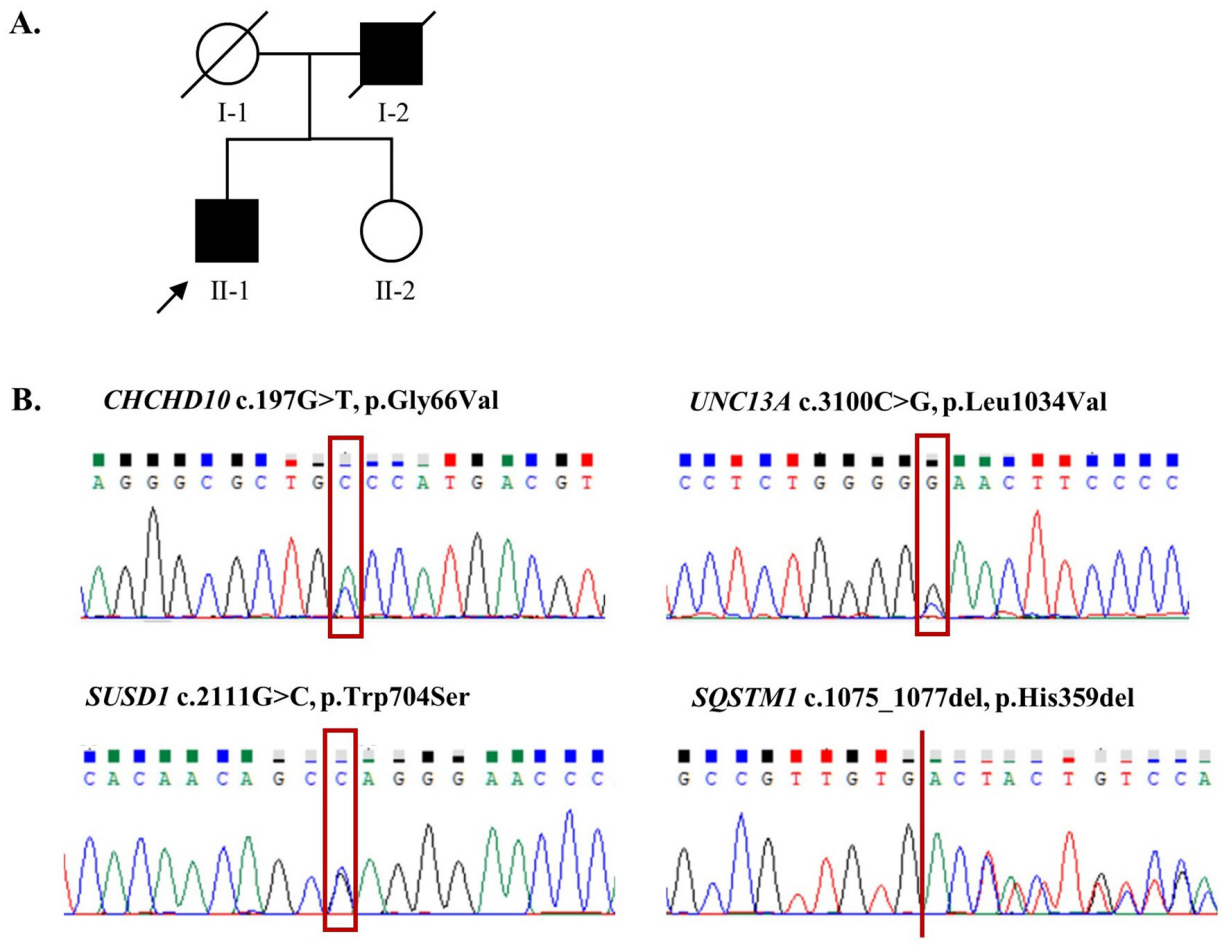
Pedigree of family 43 and the four ALS-related gene mutations identified in the proband. **(A)** Pedigree chart of Family 43. The proband of this family is marked with an arrow; filled and empty symbols indicate individuals with ALS and without ALS, respectively; circles, females; squares, males; diagonal line, deceased individual; the survival status of II-1 and II-2 is unknown due to loss of follow-up. **(B)** Sanger sequencing for the variants in CHCHD10, UNC13A, SUSUD1, and SQSTM1.

Demographic and clinical characteristics of all 127 patients included in this study are presented in Table 1. Among the 34 probands who underwent whole-genome sequencing, only one variant located in the *CHCHD10* gene, *CHCHD10* Gly66Val, was identified in the proband of Family 43 (Figure 2A). Additionally, this patient harbored three additional rare variants in other ALS-associated genes that met our variant filtering criteria: *UNC13A* p.Leu1034Val, *SUSD1* p.Trp704Ser, and *SQSTM1* p.His359del, which may have collectively contributed to the development of ALS in this pedigree (Table 2). All the variants have been verified through Sanger sequencing (Figure 2B). Multiple tools were employed to predict the pathogenicity of these variants (Table 3).

**Table 1:** Demographic and clinical characteristics of FALS patients included in this study.

	**All probands (*n* = 127)**	**Proband of family 43**
**Sex, num (%)**		
- Male	80 (63%)	Male
- Female	47 (27%)	
**Age** ^**c**^ **, years**	45.50 ± 12.45^a^	58
**Age at onset, years**	41.86 ± 13.25^a^	53
**Site of onset, num (%)**		
- Bulbar	15 (11.8%)	
- Spinal	111 (87.4%)	Spinal
- Unknown	1 (0.8%)	
**Diagnosis delay from the onset, months**	29 (13.0–69.5)^b^	71
**ALSFRS score at diagnosis**	33.47 ± 6.75^a^	39
**Progression rate** ^**d**^		
- Baseline	0.151 (0.042–0.489)^b^	0.014
- Follow-up	0.143 (0.018–0.667)^b^	0.026
**Use of medication, num (%)**	51 (40.2%)	L-carnitine, vitamin E, and coenzyme Q
**Survival, months**	54 (26–89)^b^	>147 months^e^

SD, standard deviation; IQR, interquartile range; ALSFRS, Amyotrophic lateral sclerosis function rating scale.

^a^Mean ± SD.

^b^Median (IQR).

^c^The age when the patient first visited Peking University Third Hospital.

^d^The exact length of survival of the patient is unknown due to loss of follow-up.

^e^The baseline rate of disease progression was determined by the formula “(40—‘baseline' ALSFRS score)/time from onset to ‘baseline' (months)”, and the follow-up rate of disease progression was calculated as “(‘baseline' ALSFRS score—‘last follow-up' ALSFRS score)/time interval between the time of ‘last follow-up' to the ‘baseline' time (months).”

**Table 2:** Variants detected in the proband of family 43.

**Gene**	**dbSNP ID**	**Position (GRCh38)**	**Transcript ID-Ensembl^a^**	**Transcript ID-RefSeqs^a^**	**cDNA change**	**Protein change**	**MAF in gnomAD (v4.1.0)**	**Type**
*CHCHD10*	rs730880031	Chr22:23767438	ENST00000484558.3	NM_213720.3	c.197G>T	p.Gly66Val	0.000001869	Missense
*UNC13A*	rs571398289	Chr19:17636139	ENST00000519716.7	NM_001080421.3	c.3100C>G	p.Leu1034Val	0.0001845	Missense
*SUSD1*	rs146843448	Chr9:112041911	ENST00000355396.7	-	c.2111G>C	p.Trp704Ser	0.00001177	Missense
*SQSTM1*	rs765964997	Chr5:179836592	ENST00000510187.5	-	c.1075_1077del	p.His359del	0.00003160	Deletion

dbSNP, database of single nucleotide polymorphisms; cDNA, complementary deoxyribonucleic acid; MAF, minor allele frequency.

^a^While we have matched variants to all versions of a gene's transcript depending on physical location in the annotation process, here the transcript where the effect was predicted to be the most severe transcript was presented.

**Table 3:** Pathogenicity prediction of variants detected in the proband of family 43.

**Variant**	**SIFT**	**PPH2**	**CADD phred score**	**FATHMM**	**Mutation taster**	**M-CAP**	**REVEL**	**PhyloP**	**PhastCons**
*CHCHD10* p.Gly66Val	Deleterious	Probably damaging	28.7	Tolerate	Disease causing	Deleterious	0.778	5.757	1
*UNC13A* p.Leu1034Val	Deleterious (low confidence)	Benign	23.5	Tolerate	Benign	–	0.314	7.929	1
*SUSD1* p.Trp704Ser	Tolerated (low confidence)	Benign	23.3	Tolerate	–	Deleterious	0.255	0.433	0.839
*SQSTM1* p.His359del	–	–	7.36	–	Benign	–	–	2.055	0.837

SIFT, Sorting Intolerant from Tolerant; PPH2, Polymorphism Phenotyping v2; CADD, Combined Annotation Dependent Depletion; FATHMM, Functional Analysis through Hidden Markov Models; M-CAP, Mendelian Clinically Applicable Pathogenicity; REVEL, Rare Exome Variant Ensemble Learner.

The criteria for considering mutations as pathogenic in CADD and REVEL are as follows: mutations with a CADD score >20 are regarded as pathogenic, and mutations with a REVEL score exceeding 0.5 are considered pathogenic. Some pathogenicity predictions for SQSTM1 p.His359del were not obtained (“–”) since it is not a point mutation.

In gnomAD v4.1.0 and the ExAC database, the *CHCHD10* p.Gly66Val variant is absent in the East Asian population, with a minor allele frequency (MAF) of 0.000001869 in total population. This variant led to the replace of glycine with valine at position 66 in the second exon of the *CHCHD10* gene. According to the Uniprot database, this amino acid site is highly conserved across various species. Predictions from SIFT, CADD, PPH2 Mutation, Taster, and M-cap all indicate that this variant is damaging or probably damaging. Additionally, scores from PhyloP and PhastCons suggest a high level of conservation. When considered collectively, these findings suggest that *CHCHD10* Gly66Val has potential pathogenicity in ALS.

The other variants in *UNC13A, SUSD1*, and *SQSTM1* also deserve attention. *SUSD1* p.Trp704Ser and *SQSTM1* p.His359del were rare in the East Asian population and exhibit extremely low MAFs in the gnomAD database (v4.1.0). *UNC13A* p.Leu1034Val was also rare (MAF = 0.0001845 in gnomAD v4.1.0), but the allele frequency is higher in the East Asian population (Allele Frequency = 0.005925). Considering the transcript that was predicted to have the most severe effect, these variants may impact the structure of the encoded proteins, potentially affecting their physiological functions. Combined with the predictions from *in silico* tools (Table 3), these factors together further support that all three variants passed our filtering criteria. Among them, *SQSTM1* p.His359del is recorded in the ClinVar database, classified as a variant of uncertain significance for frontotemporal dementia and/or amyotrophic lateral sclerosis 1 (FTD-ALS1) and Paget disease of bone 2, early-onset (PDB2). However, it has not been reported in the literature in individuals with *SQSTM1*-related conditions. The remaining two variants are novel and have not been documented in ClinVar. According to annotations from the InterPro database, both variants affected amino acids located within or near important structural domains. Specifically, the amino acid affected by *UNC13A* p.Leu1034Val resides in the MUN domain, which is implicated in regulating membrane trafficking, suggesting its potential role in membrane-related processes. The amino acid affected by *SUSD1* p.Trp704Ser is located near the fibronectin type 3 domain of the receptor-type tyrosine-protein phosphatase U (Fn3_RPTPU) domain of the SUSD1 protein, which is close to the transmembrane region, suggesting its potential involvement in interactions with the cell membrane or extracellular components. Based on the ACMG guidelines, we currently classify these variants as variants of uncertain significance due to insufficient evidence. However, given the complex genetic landscape and pathogenic mechanisms in ALS patients, we cannot rule out their potential involvement in ALS.

### 3.2 Clinical presentation

The patient initially presented with bilateral lower limb weakness at the age of 53, with the right lower limb being more severely affected. He subsequently encountered difficulties when ascending and descending stairs. During the next 5 years, there was minimal progression in the patient's condition. However, at the age of 58, the weakness began to spread, and numbness developed in his left foot. Physical examination revealed both upper motor neuron and lower motor neuron signs at the lumbar level. Neurophysiological testing indicated evidence of chronic denervation in the cervical, thoracic, and lumbar segments. There were no observed bulbar symptoms or cognitive impairment. The patient received L-carnitine, vitamin E, and coenzyme Q as part of his management plan. After 71 months from disease onset, the ALSFRS-R score was 39. During his last follow-up in April 2015, the patient was still alive, with an ALSFRS score of 37. He was lost to follow-up afterwards, with a total disease duration exceeding 144 months.

The proband's father is also diagnosed with ALS. According to the proband's memory, the father exhibited symptoms around the age of 50, with an onset in the lower limbs, and the progression of the disease was relatively slow. However, specific details about the illness are not available. The proband's sister does not show any ALS-related symptoms but has a history of tumors. Unfortunately, we were unable to obtain blood samples from these and other family members, preventing further pedigree segregation analysis.

## 4 Discussion

In this study, we conducted whole genome sequencing in 34 FALS probands to identify their potential pathogenic variants and explore the genotype-phenotype associations within an oligogenic context. In one proband, we identified a p.Gly66Val variant in *CHCHD10*, along with three VUS in *UNC13A, SUSD1* and *SQSTM1*. We believe that the cumulative effect of these four variants together contributes to the clinical manifestation of ALS in this patient. This finding provides evidence for potential oligogenic pathogenicity and advances our understanding of the complex clinical manifestations in *CHCHD10* variant carriers.

### 4.1 *CHCHD10* p.Gly66Val

Variants in *CHCHD10* gene have been linked to a wide spectrum of disorders including ALS, FTD, SMAJ (5), CMT2 (6) as well as myopathy (8), with a higher prevalence observed in ALS and FTD patients. In China, screenings for the *CHCHD10* gene in ALS patients have revealed a lower variant frequency compared with European populations, with variants detected only in sporadic cases (3, 26, 27).

The *CHCHD10* Gly66Val variant is commonly found in relatively mild motor neuron diseases, including late-onset spinal motor neuronopathy (spinal muscular atrophy Jokela type, SMAJ) (5, 28) and type 2 Charcot-Marie-Tooth disease (CMT2) (6), both prevalent in the Finnish population. However, this variant has not yet been reported in Chinese populations. Compared to ALS, these two diseases generally exhibit milder progression and more favorable prognosis. Although infrequently reported, a single case of this variant in ALS has been identified. In 2014, Müller et al. (29) described a familial ALS patient carrying the *CHCHD10* Gly66Val variant. Compared to classic ALS, this patient exhibited a slow-progressing, spinal-onset form of ALS with extended survival, consistent with the phenotype observed in our proband. Slow progression and longer survival are frequently reported among ALS patients with *CHCHD10* variants (29). Actually, research has indicated that the clinical phenotype associated with *CHCHD10* p.Gly66Val exhibits significant heterogeneity, with individuals carrying the same variant within the same family receiving different diagnoses (30). *CHCHD10*-related diseases show clinical overlap, and therefore comprehensive diagnostic assessments may be required for a definitive diagnosis. The complex clinical phenotypes of these patients suggest that they may not be exclusively attributable to a single genetic variant; instead, the possibility of a convergence of multiple genetic factors is implicated.

Several studies have explored the role of the p.Gly66Val variant in motor neuron disease. Using bioinformatics, homology modeling, and multiple molecular dynamics simulations, Alici et al. reported that the α-helix and β-sheet formations of wild-type *CHCHD10* are disrupted by the Gly66Val variant, affecting its overall structure (31). The Gly66Val variant leads to weaker intramolecular interactions and greater protein flexibility compared to the wild-type protein, potentially facilitating degradation by cellular machinery (31). Brockmann et al. (32) also demonstrated that *CHCHD10* p.Gly66Val exhibits an increased degradation rate and contributes to motor neuron disease via haploinsufficiency in patient-derived cell lines and an *in vivo* zebrafish model. In addition, *CHCHD10*-related mutations have also demonstrated an impact on TDP-43 pathology and mitochondrial dysfunction (33, 34). Genin et al. (34) observed that fibroblasts harboring the *CHCHD10* Ser59Leu or Gly66Val variants show comparable mitochondrial accumulation of phosphorylated TDP-43. However, mitochondrial fragmentation and cell death are significantly less pronounced in Gly66Val cells compared to Ser59Leu cells (34). This difference may explain the clinical differences between these variants: p.Ser59Leu is associated with a severe form of motor neuron disease, characterized by ataxia, dementia, and an ALS-like presentation (4), whereas p.Gly66Val is more commonly linked to a benign form of lower motor neuron disease, as mentioned above.

Therefore, based on the currently available evidence and ACMG guidelines, we tend to classify *CHCHD10* p.Gly66Val as a pathogenic variant in ALS for the following reasons: (1) Well-established *in vitro* functional studies have demonstrated a damaging effect of this variant on the gene product, as discussed above (PS3). (2) The variant has been observed in unrelated patients with similar phenotypes, including the patient described by Müller et al. and the proband in our study, and it is extremely rare in public databases (MAF = 0.000001869 in gnomAD v4.1.0) (PS4). (3) Computational evidence supports its deleterious effect on the gene or gene product, as detailed in Table 3 (PP3).

### 4.2 Three VUS identified in this proband

Studies have shown the potential impact of VUS on ALS patients, indicating that focusing solely on pathogenic mutations may not fully capture ALS's complex genetic architecture (35). Approximately 21% of ALS patients are reported to carry VUS (36). The effects of the three other rare variants detected in this proband remain uncertain compared to *CHCHD10* p.Gly66Val. However, it's worth noting that variants that are found to lack penetrance or sufficiency for the development of the disease may still act as genetic risk factors, so their potential role in ALS cannot be dismissed.

*UNC13A* is mainly expressed in neural tissues and plays a role in synaptic transmission by initiating and anchoring synaptic vesicles (37). It is a prominent candidate gene for ALS, with *UNC13A* SNPs being significant genetic markers associated with both frontotemporal dementia (FTD) and ALS in genome-wide association studies (38). These SNPs are primarily located in intronic regions. The mechanisms linking these variants to ALS may involve the loss of TDP-43 function, which leads to the inclusion of a cryptic exon in *UNC13A*, resulting in nonsense-mediated decay and loss of UNC13A protein (39, 40). In contrast, the p.Leu1034Val variant we detected is a missense variant located in exon 26 of *UNC13A*. While missense variants in *UNC13A* are not frequently reported in ALS, the p.Pro814Leu variant identified in a patient with dyskinetic movement disorder and autism has been associated with elevated initial synaptic vesicle release likelihood and abnormal short-term synaptic plasticity (41). As synaptic abnormalities are also observed in ALS (42), the pathogenic mechanism of p.Leu1034Val may also be related to this process. This variant affects an amino acid located in the MUN domain of the UNC13A protein, which contains several evolutionarily conserved domains involved in synaptic transmission, as well as synaptic vesicle docking and priming (43).

The *SUSD1* gene encodes the sushi domain-containing protein 1 precursor, known for its involvement in protein-protein interactions. This protein is believed to raise the risk of venous thromboembolism by interacting with factors in the coagulation pathway via its sushi domain (44). In 2007, a whole-genome gene association study on sporadic ALS patients initially established an association between *SUSD1* and ALS (45). However, the specific impact of single-site variants on disease risk remains uncertain. Further studies revealed an upregulated circular RNA within this gene in ALS patients' white blood cells, potentially serving as a biomarker (46). While the variant we detected provides direct evidence of *SUSD1*'s involvement in ALS, its functional effect requires further investigation.

The *SQSTM1* gene encodes p62, a protein crucial for protein degradation via both proteasome and autophagy pathways. SQSTM1-positive inclusions often co-localize with ubiquitin and *TARDBP*, and are commonly found in ALS and FTD patients (47). Since 2011, several *SQSTM1* variants have been identified in ALS patients (48, 49), with lower frequencies reported in Chinese populations (50). Some *SQSTM1* variants are also linked to Paget's disease of bone (PDB), indicating potential interactions with other genetic or environmental factors influencing ALS risk or disease phenotype (48). The *SQSTM1* variant we identified results in the deletion of histidine at position 359 in the protein, with a MAF of 0.00003160 in the gnomAD database. In addition to the existing records in ClinVar, our report provides new evidence supporting the association of this variant with ALS. Further functional studies are needed to evaluate its impact on protein structure or function.

### 4.3 Oligogenic pathogenesis as a potential explanation for complex phenotypes in *CHCHD10* Gly66Val carriers

The phenotypic complexity seen in carriers of *CHCHD10* variants were similar to that of *C9orf72*. Initially identified in ALS-FTD, *C9orf72* has since been associated with a wide range of neurodegenerative and psychiatric conditions, including Alzheimer's, Parkinson's, Huntington-like phenotypes, and schizophrenia (11). One hypothesis explaining the variability in carriers of *C9orf72* variants is that the existence additional genetic variants may influence the phenotype, supported by evidence for an oligogenic model (11). A similar explanation could apply to *CHCHD10* variants, where the combined contribution of multiple genetic variants could lead to the diverse clinical presentations.

ALS is considered to arise through a multistep process involving both genetic and environmental factors (51). Given the oligogenic pathogenesis of ALS and the characteristics of the *CHCHD10* p.Gly66Val discussed previously, we suggest that this variant alone may not be adequate to trigger ALS. Instead, it could be the interaction between different genetic variants that determined the final ALS phenotype, rather than showing the relatively mild phenotypes associated with *CHCHD10* p.Gly66Val. The cumulative genetic burden from ALS-related genes might increase the patient's risk of developing a more severe form of the disease, as other studies on oligogenic ALS have shown (14, 52). This could help explain the variation in clinical phenotypes among patients with the same mutation. However, further research is needed to clarify how these variants contribute to ALS.

For the proband reported in this study, it is possible that the onset of ALS is due to the combined effect of *CHCHD10* p.Gly66Val and the VUS in three other genes. In contrast, the Finnish ALS patient carrying *CHCHD10* p.Gly66Val, as reported by Müller et al. (29), did not have any additional ALS-related gene variants detected through whole exome sequencing at the time. However, with new ALS-related genes being discovered in recent studies, it is challenging to completely rule out the possibility of other contributing variants. Larger-scale genetic screenings in broader populations may help validate this hypothesis.

### 4.4 Phenotypic consequences of the four combined rare variants

The four variants carried by this proband have not been frequently reported to co-occur with other ALS-related gene variants. This may be partly explained by the lack of comprehensive genetic screening or stricter variant filtering criteria. One study reported the co-occurrence of *CHCHD10* and *C9orf72* variants in an ALS-FTD patient (53), and another reported a person carrying both *TBK1* and *SQSTM1* variants, who was diagnosed with non-fluent variant primary progressive aphasia (nfv-PPA) (54). However, the specific phenotypic characteristics of these patients remain unclear. Consequently, the simultaneous presence of these four rare gene variants in a single patient makes our case particularly remarkable.

While previous research often associates an increased burden of rare variants with reduced survival (10, 36), our proband's clinical characteristics deviated from this pattern, showing slower disease progression and extended survival compared to the cohort's average. The inconsistency may be attributed to the fact that the clinical phenotype of patients with multiple gene variants represent statistical patterns, which differs by individual cases depending on the combined effect of the exact variants. Interestingly, this patient's clinical phenotype aligned with typical features of ALS patients carrying *CHCHD10* variants. We propose that the cumulative genetic burden of the four variants contributed to the development and specific phenotype of ALS, with *CHCHD10* p.Gly66Val playing the main role, and the other three VUS potentially acting as modifying factors. Additionally, the presence of multiple variants may have an impact on the age of disease onset, as some *CHCHD10* variant carriers in China showed a later average onset (27). However, we cannot yet draw definitive conclusions regarding the exact effects of these variants in this patient currently.

### 4.5 Limitations

Our study has several limitations. First, due to the high mortality rate of ALS, we were unable to obtain blood samples from additional family members, which prevented us from performing segregation analysis. Second, the small sample size and rarity of these gene variants meant that variants in these four genes were identified in only one proband. As a result, we cannot provide compelling genetic or statistical evidence for the proposed oligogenic pathogenic mechanism related to *CHCHD10* Gly66Val. Currently, no definitive conclusions can be drawn regarding the pathogenicity of the three variants in *UNC13A, SQSTM1*, and *SUSD1*. We plan to conduct functional studies using cell or animal models to further clarify the roles of these variants and their interactions. At this stage, this study primarily reports the screening results for the *CHCHD10* gene in the Chinese FALS population. While we have not extensively explored the pathogenic mechanisms of each variant, our hypothesis that *CHCHD10* Gly66Val is involved in ALS through interactions with other variants is reasonable, given previous reports on oligogenic mechanisms in ALS. This also provides insights into the complex phenotypes associated with *CHCHD10* variants. Future screenings in larger cohorts will help provide more robust evidence for this hypothesis.

### 4.6 Conclusion

In summary, our study identified the *CHCHD10* p.Gly66Val variant in Chinese FALS patients for the first time, and revealed its coexistence with three VUS in other ALS-related genes (*UNC13A* p.Leu1034Val, *SUSD1* p.Trp704Ser, and *SQSTM1* p.His359del) through whole-genome sequencing. We suppose that the interplay between these variants led to typical ALS symptoms in this patient, rather than other milder neuromuscular disorders caused by *CHCHD10* p.Gly66Val. Our research further supports the oligogenic pathogenesis of ALS. Future investigations focused on unraveling the interactions between these genetic variants can deepen our understanding of the intricate mechanisms underlying ALS.

## Data Availability

The original contributions presented in the study are included in the article and the Supplementary material; further inquiries can be directed to the corresponding author.
