# Supplementary material for: Combined impact of CHCHD10 p.Gly66Val and three other variants suggests oligogenic contributions to ALS
Source: Front Neurol. 2025 Mar 18;16:1438207. doi: 10.3389/fneur.2025.1438207 (PMC11959081; doi:10.3389/fneur.2025.1438207)
Supplement: Supplementary file 1 [file Table_1.docx]

**Supplementary Materials**

**Supplementary Table 1. 113 rare genes related to ALS from databases and papers**

| Gene symbol | Gene name | Classification | Known inheritance pattern | pHaplo | pTriplo | Source |
| --- | --- | --- | --- | --- | --- | --- |
| *ALS2* | alsin Rho guanine nucleotide exchange factor ALS2 | causative gene | AR | 0.8 | 0.52 | ALSoD |
| *ANG* | angiogenin | causative gene | AD* | 0.15 | 0.19 | ALSoD |
| *ANXA11* | annexin A11 | causative gene | AD | 0.21 | 0.27 | ALSoD |
| *C9orf72* | C9orf72-SMCR8 complex subunit | causative gene | AD | 0.37 | 0.15 | ALSoD |
| *CCNF* | cyclin F | causative gene | AD | 0.77 | 0.36 | ALSoD |
| *CHCHD10* | coiled-coil-helix-coiled-coil-helix domain containing 10 | causative gene | AD | 0.2 | 0.19 | ALSoD |
| *CHMP2B* | charged multivesicular body protein 2B | causative gene | AD | 0.11 | 0.22 | ALSoD |
| *ERBB4* | erb-b2 receptor tyrosine kinase 4 | causative gene | AD | 0.97 | 0.79 | ALSoD |
| *FIG4* | FIG4 phosphoinositide 5-phosphatase | causative gene | AD | 0.24 | 0.32 | ALSoD |
| *FUS* | FUS RNA binding protein | causative gene | AD | 0.9 | 0.94 | ALSoD |
| *HNRNPA1* | heterogeneous nuclear ribonucleoprotein A1 | causative gene | AD | 0.92 | 0.88 | ALSoD |
| *MATR3* | matrin 3 | causative gene | AD | 0.97 | 0.99 | ALSoD |
| *OPTN* | optineurin | causative gene | AD, AR | 0.38 | 0.6 | ALSoD |
| *PFN1* | profilin 1 | causative gene | AD | 0.95 | 0.7 | ALSoD |
| *SETX* | senataxin | causative gene | AD | 0.97 | 0.88 | ALSoD |
| *SIGMAR1* | sigma non-opioid intracellular receptor 1 | causative gene | AR | 0.22 | 0.62 | ALSoD |
| *SOD1* | superoxide dismutase 1 | causative gene | AD, AR | 0.75 | 0.68 | ALSoD |
| *SPG11* | SPG11 vesicle trafficking associated, spatacsin | causative gene | AR | 0.21 | 0.32 | ALSoD |
| *SQSTM1* | sequestosome 1 | causative gene | AD | 0.51 | 0.97 | ALSoD |
| *TARDBP* | TAR DNA binding protein | causative gene | AD | 0.97 | 0.91 | ALSoD |
| *TBK1* | TANK binding kinase 1 | causative gene | AD | 0.49 | 0.8 | ALSoD |
| *TIA1* | TIA1 cytotoxic granule associated RNA binding protein | causative gene | AD | 0.71 | 0.85 | ALSoD |
| *TUBA4A* | tubulin alpha 4a | causative gene | AD | 0.47 | 0.86 | ALSoD |
| *UBQLN2* | ubiquilin 2 | causative gene | XLD | NA | NA | ALSoD |
| *VCP* | valosin containing protein | causative gene | AD | 0.93 | 1 | ALSoD |
| *CYLD* | *CYLD* lysine 63 deubiquitinase | causative gene | AD | 0.99 | 0.98 | ALSoD |
| *SPTLC1* | serine palmitoyltransferase long chain base subunit 1 | causative gene | AD | 0.27 | 0.11 | Pubmed (PMID: 34459874) |
| *WDR7* | WD repeat domain 7 | causative gene | unknown | 0.94 | 0.59 | Pubmed (PMID: 32750315) |
| *CFAP410* | cilia and flagella associated protein 410 | causative gene | AD | NA | NA | ALSoD |
| *ALS3* | - | loci associated with ALS | AD | NA | NA | ALSoD |
| *ALS7* | - | loci associated with ALS | unknown | NA | NA | ALSoD |
| *PPARGC1A* | PPARG coactivator 1 alpha | MGI-associated gene | unknown | 0.96 | 0.8 | The MGI Database |
| *TFAM* | transcription factor A, mitochondrial | MGI-associated gene | unknown | 0.53 | 0.4 | The MGI Database |
| *TXNRD1* | thioredoxin reductase 1 | MGI-associated gene | unknown | 0.59 | 0.64 | The MGI Database |
| *ADARB1* | adenosine deaminase RNA specific B1 | MGI-associated gene | AR | 0.89 | 0.9 | The MGI Database |
| *PSMC4* | proteasome 26S subunit, ATPase 4 | MGI-associated gene | unknown | 0.43 | 1 | The MGI Database |
| *ZNF106* | zinc finger protein 106 | MGI-associated gene | unknown | 0.56 | 0.68 | The MGI Database |
| *EPG5* | ectopic P-granules 5 autophagy tethering factor | MGI-associated gene | AR | 0.65 | 0.39 | The MGI Database |
| *ATXN2* | ataxin 2 | susceptibility to ALS | AD | 0.98 | 0.96 | ALSoD |
| *DCTN1* | dynactin subunit 1 | susceptibility to ALS | AD, AR | 0.36 | 0.93 | ALSoD |
| *KIF5A* | kinesin family member 5A | Causative gene | AD | 0.49 | 0.92 | ALSoD |
| *NEFH* | neurofilament heavy | susceptibility to ALS | AD, AR | 0.29 | 0.51 | ALSoD |
| *NEK1* | NIMA related kinase 1 | susceptibility to ALS | AD | 0.81 | 0.48 | ALSoD |
| *PRPH* | peripherin | susceptibility to ALS | AD, AR | 0.15 | 0.06 | ALSoD |
| *GRB14* | growth factor receptor bound protein 14 | susceptibility to ALS | unknown | 0.44 | 0.34 | ALSoD |
| *MAPT* | microtubule associated protein tau | susceptibility to ALS | AR | 0.3 | 0.18 | ALSoD |
| *SOD2* | superoxide dismutase 2 | susceptibility to ALS | unknown | 0.58 | 0.33 | ALSoD |
| *TRPM7* | transient receptor potential cation channel subfamily M member 7 | susceptibility to ALS | AD | 0.9 | 0.24 | ALSoD |
| *CNTN6* | contactin 6 | Modify gene | unknown | 0.15 | 0.05 | ALSoD |
| *UNC13A* | unc-13 homolog A | Modify gene | AD | 0.76 | 0.95 | ALSoD |
| *CX3CR1* | C-X3-C motif chemokine receptor 1 | Modify gene (lifetime and disease progress) | unknown | 0.1 | 0.16 | ALSoD |
| *CAMTA1* | Calmodulin brinding transcriptions activator 1 | Modify gene (lifetime) | AD | 1 | 1 | ALSoD |
| *PON1* | paraoxonase 1 | Modify gene (lifetime) | unknown | 0.25 | 0.31 | ALSoD |
| *SPAST* | spastin | Modify gene (lifetime) | AD | 0.98 | 0.81 | ALSoD |
| *ENAH* | ENAH actin regulator | Modify gene (disease development) | AR | 0.98 | 0.96 | ALSoD |
| *OGG1* | 8-oxoguanine DNA glycosylase | Modify ( respiratory system) | AR,AD | 0.59 | 0.55 | ALSoD |
| *PNPLA6* | patatin like phospholipase domain containing 6 | associated-*FUS*ion gene | unknowm | 0.24 | 0.84 | ALSoD |
| *DOC2B* | double C2 domain beta | associated-therapy | AR | 0.82 | 0.22 | ALSoD |
| *LOX* | lysyl oxidase | associated-therapy | AR | 0.89 | 0.80 | ALSoD |
| *VEGFA* | vascular endothelial growth factor A | associated-therapy | unknown | 0.58 | 0.45 | ALSoD |
| *CNTN4* | contactin 4 | Strong evidence | AR | 0.7 | 0.18 | ALSoD |
| *CRIM1* | cysteine rich transmembrane BMP regulator 1 | Strong evidence | AR,AD | 0.98 | 0.58 | ALSoD |
| *CYP2D6* | Cytochrome P450 family 2 subfamily D member 6 | Strong evidence | unknown | 0.38 | 0.35 | ALSoD |
| *KDR* | kinase insert domain receptor | Strong evidence | unknown | 0.89 | 0.99 | ALSoD |
| *AR* | androgen receptor | Moderate evidence | AR,AD | 0.09 | 0.28 | ALSoD |
| *ARHGEF28* | rho guanine nucleotide exchange factor 28 | Moderate evidence | unknown | 0.72 | 0.82 | ALSoD |
| *ARPP21* | cAMP Regulated Phosphoprotein 21 | Tenuous (ALSoD) | unknown | 0.27 | 0.1 | ALSoD |
| *ATXN1* | ataxin 1 | Moderate evidence | AD | 0.99 | 0.94 | ALSoD |
| *CCS* | copper chaperone for superoxide dismutase | Moderate evidence | unknown | 0.12 | 0.62 | ALSoD |
| *CDH13* | cadherin 13 | Moderate evidence | AD | 0.84 | 0.25 | ALSoD |
| *CDH22* | cadherin 22 | Moderate evidence | unknown | 0.79 | 0.81 | ALSoD |
| *APEX1* | apurinic/apyrimidinic endodeoxyribonuclease 1 | Tenuous (ALSoD) | unknown | 0.51 | 0.56 | ALSoD |
| *DISC1* | DISC1 scaffold protein | Tenuous (ALSoD) | XLR | 0.77 | 0.43 | ALSoD |
| *DPP6* | dipeptidyl peptidase like 6 | Tenuous (ALSoD) | unknown | 0.82 | 0.12 | ALSoD |
| *DYNC1H1* | dynein cytoplasmic 1 heavy chain 1 | Tenuous (ALSoD) | unknown | 0.98 | 1 | ALSoD |
| *EFEMP1* | EGF containing fibulin extracellular matrix protein 1 | Tenuous (ALSoD) | AD | 0.81 | 0.55 | ALSoD |
| *EWSR1* | EWS RNA binding protein 1 | Tenuous (ALSoD) | AD | 0.99 | 0.99 | ALSoD |
| *FEZF2* | FEZ family zinc finger 2 | Tenuous (ALSoD) | unknown | 0.97 | 0.97 | ALSoD |
| *GLT8D1* | glycosyltransferase 8 domain containing 1 | Tenuous (ALSoD) | AR | 0.25 | 0.43 | ALSoD |
| *GRN* | granulin precursor | Tenuous (ALSoD) | AD | 0.16 | 0.34 | ALSoD |
| *HEXA* | hexosaminidase subunit alpha | Tenuous (ALSoD) | AD | 0.44 | 0.5 | ALSoD |
| *HFE* | homeostatic iron regulator | Tenuous (ALSoD) | AD | 0.21 | 0.16 | ALSoD |
| *ITPR2* | inositol 1,4,5-triphosphate receptor type 2 | Tenuous (ALSoD) | unknown | 0.85 | 0.73 | ALSoD |
| *KIFAP3* | kinesin associated protein 3 | Tenuous (ALSoD) | unknown | 0.34 | 0.54 | ALSoD |
| *LMNB1* | lamin B1 | Tenuous (ALSoD) | AD | 0.74 | 0.29 | ALSoD |
| *NEFL* | neurofilament light | Tenuous (ALSoD) | AD | NA | NA | ALSoD |
| *ODR4* | odr-4 GPCR localization factor homolog | Tenuous (ALSoD) | AD | NA | NA | ALSoD |
| *PLEKHG5* | pleckstrin homology and RhoGEF domain containing G5 | Tenuous (ALSoD) | AR,AD | 0.71 | 0.63 | ALSoD |
| *PON2* | paraoxonase 2 | Tenuous (ALSoD) | unknown | 0.32 | 0.33 | ALSoD |
| *RAMP3* | receptor activity modifying protein 3 | Tenuous (ALSoD) | AR | 0.19 | 0.24 | ALSoD |
| *SARM1* | sterile alpha and TIR motif containing 1 | Tenuous (ALSoD) | AR | 0.17 | 0.81 | ALSoD |
| *SCN7A* | sodium voltage-gated channel alpha subunit 7 | Tenuous (ALSoD) | unknown | 0.69 | 0.32 | ALSoD |
| *SEMA6A* | semaphorin 6A | Tenuous (ALSoD) | unknown | 0.98 | 0.94 | ALSoD |
| *SLC39A11* | solute carrier family 39 member 11 | Tenuous (ALSoD) | unknown | 0.5 | 0.15 | ALSoD |
| *SLC52A3* | solute carrier family 52 member 3 | Tenuous (ALSoD) | unknown | 0.43 | 0.1 | ALSoD |
| *SPG7* | SPG7 matrix AAA peptidase subunit, paraplegin | Tenuous (ALSoD) | AR | 0.52 | 0.18 | ALSoD |
| *SS18L1* | SS18L1 subunit of BAF chromatin remodeling complex | Tenuous (ALSoD) | unknown | 0.82 | 0.9 | ALSoD |
| *SUSD1* | sushi domain containing 1 | Tenuous (ALSoD) | AR,AD | 0.17 | 0.37 | ALSoD |
| *SYNE1* | spectrin repeat containing nuclear envelope protein 1 | Tenuous (ALSoD) | unknown | 0.87 | 1 | ALSoD |
| *TAF15* | TATA-box binding protein associated factor 15 | Tenuous (ALSoD) | AR,AD | 0.23 | 0.69 | ALSoD |
| *TNIP1* | TNFAIP3 interacting protein 1 | Tenuous (ALSoD) | unknown | 0.71 | 0.48 | ALSoD |
| *VRK1* | VRK serine/threonine kinase 1 | Tenuous (ALSoD) | AR | 0.75 | 0.56 | ALSoD |
| *ZFP64* | ZFP64 zinc finger protein | Tenuous (ALSoD) | unknown | 0.88 | 0.4 | ALSoD |
| *ZNF512B* | zinc finger protein 512B | Tenuous (ALSoD) | unknown | 0.99 | 0.96 | ALSoD |
| *AGT* | angiotensinogen | Tenuous (ALSoD) | AR | 0.43 | 0.16 | ALSoD |
| *DIAPH3* | diaphanous related formin 3 | conflicting | unknown | 0.32 | 0.15 | ALSoD |
| *DNAJC7* | DnaJ heat shock protein family (Hsp40) member C7 | conflicting | unknown-conflicting | 0.8 | 1 | ALSoD |
| *NIPA1* | NIPA magnesium transporter 1 | conflicting | unknown | 0.11 | 0.53 | ALSoD |
| *RBCK1* | RANBP2-type and C3HC4-type zinc finger containing 1 | weakness evidence | AR | 0.94 | 0.53 | Pubmed (PMID: 36726375,35563729,27552911) |
| *TRIB3* | tribbles pseudokinase 3 | weakness evidence | unknown | 0.63 | 0.11 | Google Scholar |
| *SNPH* | syntaphilin | weakness evidence | unknown | 0.93 | 0.45 | Pubmed (PMID: 34193962,28472658) |
| *CDC25B* | cell division cycle 25B | weakness evidence | unknown | 0.78 | 1 | Pubmed (PMID: 25867286) |
| *TGM6* | transglutaminase 6 | weakness evidence | AD | NA | NA | Pubmed (PMID: 26144268) |

*Gene symbol and Gene name were checked on the web of HGNC (http://www.genenames.org). AD: autosomal dominant. AR: autosomal recessive. XLR: chromosome X linked recessive. pHaplo: Predicted Probability of Haploinsufficiency, pTriplo: Predicted Probability of Triplosensitivity (http://www.deciphergenomics.org).

**Supplementary Table 2. PCR primers for amplification in Sanger sequencing**

| **Gene** | **Left Primer** | **Right Primer** |
| --- | --- | --- |
| CHCHD10 | TTAACCCTGCTTCCTCCCAC | GGAAGCCTGCCTCTAAGTGA |
| UNC13A | GACCCACAATTACCCCCAGG | TCCACTGTCCATTTCAGGCC |
| SUSD1 | CCCAGCTGTTCTTCGTGACT | ATGGATTGAGCACAGTGGGG |
| SQSTM1 | CATGGCTTCCTTACTGTTTC | ACCAGGTCTGTAGCAGAAAA |
